# Association of plasma propionate concentration with coronary artery disease in a large cross-sectional study

**DOI:** 10.3389/fcvm.2023.1063296

**Published:** 2023-02-01

**Authors:** Nikolaos Pagonas, Felix S. Seibert, Gerhard Liebisch, Maximillian Seidel, Theodoros Giannakopoulos, Benjamin Sasko, Oliver Ritter, Nina Babel, Timm H. Westhoff

**Affiliations:** ^1^Department of Cardiology, Brandenburg Medical School Theodor Fontane, Brandenburg an der Havel, Germany; ^2^Faculty of Health Sciences, Joint Faculty of the Brandenburg University of Technology Cottbus – Senftenberg, The (MHB) Theodor Fontane and the University of Potsdam, Potsdam, Germany; ^3^Medical Department I, Marien Hospital Herne, Ruhr-University of Bochum, Bochum, Germany; ^4^Institute of Clinical Chemistry and Laboratory Medicine, University Hospital Regensburg, Regensburg, Germany; ^5^Department of Cardiology, Knappschaftskrankenhaus Bottrop, Academic Teaching Hospital, University of Duisburg-Essen, Duisburg, Germany

**Keywords:** short chain fatty acids, coronary artery disease, propionate, atherosclerosis, microbiome

## Abstract

**Background:**

Microbiome has been linked to the pathogenesis of coronary artery disease (CAD) but data providing direct evidence for an association of short-chain fatty acids (SCFA) with CAD are lacking. This study aimed to evaluate the role of propionate, the most important SCFA in patients with CAD.

**Methods:**

We performed a cross-sectional study enrolling patients admitted for invasive coronary angiography in two university hospitals in Germany. Patients with known or suspected CAD and risk factors for cardiovascular disease were prospectively recruited. Blood sampling was performed after overnight fasting and before invasive procedures. Measurement of propionate was performed by liquid chromatography.

**Results:**

The study included 1,253 patients (median [IQR], 67 [58–76] years; 799 men [64%]). A total of 739 had invasively confirmed CAD with at least one coronary artery stenosis ≥50% and 514 had exclusion of CAD. CAD patients had significant lower levels of propionate (median 5.75 μM, IQR, 4.1–7.6) compared to the non-CAD groups 6.53 μM (4.6–8.6, *p* < 0.001). Multivariate linear regression analysis revealed an odds ratio of 0.94 (CI 0.90–0.98, *p* = 0.002) for propionate as predictor of CAD. The odds ratio was further decreased to 0.45 (CI 0.31–0.65, *p* < 0.001) when comparing patients in the lowest quartile of propionate with those with higher levels of propionate.

**Conclusion:**

The study provides large-scale *in vivo* data for the association of propionate to manifest coronary artery disease, independent of other traditional cardiovascular risk factors.

## Introduction

Alterations of the gut microbiome have been linked to cardiovascular disease including coronary artery disease (CAD) in cross sectional studies. The microbiome metabolizes resistant dietary fibers through fermentation to short chain fatty acids (SCFA) including propionate, butyrate, and acetate, which can pass the mucosal border and thereby enter the circulation. There is accumulating evidence from animal studies that propionate and butyrate elicit favorable effects on endothelium, lipid profile, blood pressure, and act as anti-inflammatory mediators (1). Propionate may improve endothelial function by activation of GPR41, reduces renin secretion, lowers serum cholesterol concentrations and elicits anti-inflammatory effects by an increase or regulatory T cells (2–5). A broad variety of therapeutic interventions including probiotics, antibiotics, stool transplantation, or supplementation of SCFA could be envisioned to increase SCFA concentrations in the circulation. The majority of these interventions are broadly available, easy to perform and are therefore promising candidates to prevent atherosclerosis.

Recently, oral supplementation of propionate led to reduction of low-density lipoprotein in patients with hypercholesterinemia (6). In an animal model, Haghikia et al. (6) demonstrated an improvement of aortic atherosclerotic lesions in response to propionate in apolipoprotein E−/− (Apoe−/−) mice fed with high-fat diet. However, human studies on the impact of SCFS concentrations on atherosclerosis are lacking (7).

We hypothesized that higher concentrations of propionate are associated with a lower risk for coronary artery disease. We therefore investigated for the first time, whether the plasma concentration of propionate is associated with the prevalence of manifest coronary artery disease in individuals undergoing coronary angiography.

## Methods

### Study design

Patients admitted for elective coronary angiography to the university hospitals of Brandenburg an der Havel and Ruhr-University Bochum, both in Germany. Were eligible for inclusion in the study. 1,253 participants were prospectively recruited between 2017 and 2020. Exclusion criteria were emergency admissions, known cancer disease, acute infectious disease (symptoms of infection or fever), known rheumatic or other inflammatory disease, and age < 18 years. Blood samples were obtained in all patients after overnight fasting and before any invasive procedure. Based on the findings of angiography patients were assigned to the CAD group (*n* = 739), if there was an at least 50% stenosis of a coronary artery. Patients in the non-CAD group (514) had no stenosis or stenosis <50% in all coronary arteries. Written informed consent was obtained from all participants. The study was approved by the local ethics committees of the medical association of Brandenburg (Nr. AS69bB/2016) and of the Ruhr University of Bochum (Nr. 15–5279) in accordance with the Declaration of Helsinki.

### Biomarker and laboratory assessment

Serum was prepared from blood samples and was cryopreserved (−80°C). Laboratory parameters were obtained by standard clinical assays in the central laboratory unit of the university hospitals.

SCFA analysis was performed as described previously by liquid chromatography tandem mass spectrometry (LC–MS/MS) upon derivatization to 3-nitrophenylhydrazones (3NPH) with minor modifications (8). Sample preparation was carried out as described for portal vein plasma (9). In brief, 10 μL of an internal standard mixture containing D5-propionic acid (Cambridge Isotope Laboratories, United States) was added to 10 μL serum and mixed. 100 μl acetonitrile were added and centrifuged after mixing. 50 μl of the supernatant were derivatized to 3NPH derivatives (8) and 10 μL of the quenched reaction mix were injected into the LC–MS/MS system.

### Statistical analysis

Normal distribution of data was checked using the Shapiro–Wilk test. In case of normal distribution, the comparison of two groups was performed with the *t*-test (>2 groups: F test). Otherwise, the Mann–Whitney-U test (2 groups) or Kruskal-Wallis test (>2 groups) was applied. Data are presented as median with interquartile range. The Chi-square test was used to compare the frequency of a categorical variable between independent groups. Pearson’s correlation coefficient assessed the correlation between two continuous variables. Logistic univariate and multivariate logistic regression analysis was used to assess the relationship between propionate and CAD b < adjusting for different risk factors. All statistical analyses were performed by using the SPSS and the GraphPad software.

## Results

### Demographic characteristics of study participants

A total of 1,253 participants were included in the analysis (*n* = 739 in the CAD group, *n* = 514 in the non-CAD group). The characteristics of the study participants are shown in Table 1. The incidence of CAD was higher in participants who were male, had diabetes, hypertension and a history of smoking compared to participants who were female or without the respective risk factor (*p* < 0.05 for all comparisons).

**Table 1:** Description of the main cardiovascular risk factors and biomarkers based on the study group.

	CAD	No CAD	*p*-value
*N*	739	514	
Age, years	69 (61–76)	63 (55–73)	<0.001
Male, no (%)	562 (76)	237 (46)	<0.001
Hypertension, no (%)	656 (89)	374 (67)	<0.001
Diabetes, no (%)	263 (36)	80 (16)	<0.001
Current smoking, no (%)	256 (35)	104 (20)	0.001
Statins, no (%)	585 (79)	174 (34)	<0.001
BMI, kg/m^2^	28 (26–32)	28 (25–32)	0.43
HDL, mg/dL	46 (39–59)	56 (45–66)	<0.001
LDL, mg/dL	102 (78–133)	126 (100–152)	<0.001
Cholesterol, mg/dL	171 (143–205)	196 (172–225)	<0.001
Triglyceride, mg/dL	128 (95–183)	116 (86–165)	0.001
Lipoprotein-a nmol/L	15 (7–82)	12 (6–34)	0.07
hsCRP, mg/dL	0.11 (0.0–0.62)	0.11 (0.0–0.86)	0.44
HbA1c, %	5.9 (5.8–6.8)	5.6 (5.3–5.9)	<0.001
Propionate, μM	5.75 (4.1–7.6)	6.53 (4.6–8.6)	<0.001

Data are presented as median with interquartile range or number (%). Statistical comparison was performed by Chi square test for categorical variables and Mann Whitney test for metric variables. Significant values are shown in bold numbers. NA, not applicable; BMI, body mass index; CAD, coronary artery disease; HbA1c, glycated hemoglobin A1c; HDL, high-density lipoprotein; hsCRP, high sensitive C reactive protein; LDL, low-density lipoprotein.

### Associations of propionate concentrations with established risk factors for coronary artery disease

In a first approach the associations of serum propionate concentrations with lipid concentrations, body mass index (BMI), Lipoprotein (a) [LP (a)], and hemoglobin A1c (HbA1c) were analyzed. In the overall study population males and persons with hypertension had lower propionate concentrations (*p* < 0.05 for all comparisons, Supplementary Table 1). Among all participants, higher age, male gender and smoking were weakly associated with lower propionate concentrations in the multivariate regression analysis (*p* < 0.05). Diabetes and triglycerides were associated with higher propionate concentrations (*p* < 0.05). In patients with CAD diabetes, triglycerides and cholesterol were still associated with higher propionate concentrations but all associations were very weak (Supplementary Table 2).

Since statins may affect the gut microbiome (10) and consequently SCFA concentrations, we performed an additional analysis by dividing patients in those with and without statins (Supplementary Table 3). Participants on statins had lower propionate concentrations compared to the group without statins (*p* < 0.001).

### Association of propionate concentrations with coronary artery disease

**Figure 1:**
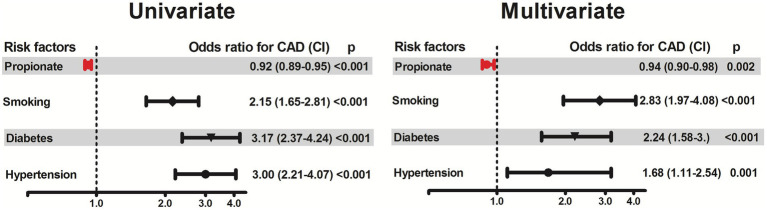
Association of propionate concentrations with the presence of coronary artery disease (CAD). The association of propionate with CAD was assessed by logistic regression using univariate and multivariate models adjusted for selected cardiovascular risk factors (hypertension, diabetes, smoking), age, gender, body-mass index and intake of statins. Odds ratios and 95% confidence intervals are presented. Odds ratios for continuous variables are per 1-SD increase.

**Figure 2:**
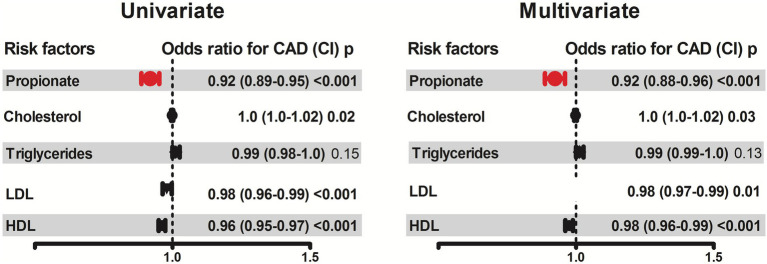
Association of propionate concentrations with the presence of coronary artery disease (CAD). The association of propionate with CAD was assessed by logistic regression using univariate and multivariate models adjusted for the major lipid parameters (HDL, LDL, Cholesterol, Triglycerides) in addition to age, gender, hypertension, diabetes and smoking. Odds ratios and 95% confidence intervals are presented. Odds ratios for continuous variables are per 1-SD increase.

Patients with CAD had lower concentrations of propionate compared to patients without CAD (*p* < 0.001, Table 1). In univariate logistic regression analysis of all study participants, an increase in 1 SD of propionate concentration was associated with 95% reduced risk for CAD [odds ratio 0.92, 95% confidence interval (CI), 0.89 to 0.95, *p* < 0.001, Figure 1]. In multivariate analyses adjusting for age, gender, diabetes, hypertension, smoking and intake of statins the odds ratio remained significant (OR 0.94, CI 95% 0.90–0.98) suggesting that propionate is an independent predictor of CAD. In a further regression analysis adjusted for HDL, LDL, cholesterol and triglycerides propionate was constantly associated with CAD (Figure 2).

The significance of propionate as a robust marker for the presence of CAD was further investigated by performing a logistic regression analysis using quartiles of propionate concentrations. Higher levels of propionate (quartile 4) were associated with a significantly lower odds ratio of 0.448, CI 95% 0.31–0.65, compared to propionate concentrations in quartile 1 (Supplementary Table 4).

## Discussion

The present cross-sectional study provides first evidence that higher concentrations of propionate are indeed associated with a lower risk of CAD independent of established cardiovascular risk factors. As described above, there is an increasing amount of experimental data indicating that propionate may elicit beneficial effects on cardiovascular risk factors like hypertension, endothelial dysfunction, and hypercholesterolemia (1, 2, 6). Whereas alterations in the microbiome have successfully been linked to a modification of cardiovascular risk, a proof that these effects might translate into a measurable reduction of CAD prevalence was missing so far.

Multivariate regression analyses revealed a negative association of propionate concentrations with the presence of CAD, which remained significant after adjustment for several risk factors. Hence, an independent vasoprotective effect of these SCFA may be postulated.

The lower concentrations of propionate in patients on statins (Supplementary Table 3) may not reflect a direct effect of statins but rather the burden of risk factors leading to administration of statins. Statins are known to modify the composition of the gut microbiome (10). Noteworthy, however, the observed association between propionate and the prevalence of CAD remained significant after adjustment for statin use as well. Due to its non-interventional, cross-sectional character, the present study is not able to attribute causality to the observed associations. Nevertheless, these first-in-human results are a crucial prerequisite for interventional studies on this promising cardiovascular prevention strategy.

Beyond its role as a metabolite of the gut microbiome, propionate is part of several food products including cheese, coffee, or seafood. Moreover, it is available as a dietary supplement, which allows supplementation independent of modifying the microbiome. We have previously shown that the achieved serum concentrations are able to elicit an immunomodulation with increases of regulatory T cells (4). So far, there are no known side effects of this dietary supplement.

## Limitations

These results are hypothesis generating and do not provide any causal association for the role of short fatty acids in the pathogenesis of atherosclerosis and coronary artery disease. Further experimental studies are warranted to shed light on the mechanism leading to an association of propionate with the coronary artery disease.

## Conclusion

Based on the present findings, future studies should investigate this new approach in primary and secondary cardiovascular prevention. After decades of focusing on traditional cardiovascular risk factors, modification of SCFA concentrations may offer new opportunities to reduce cardiovascular morbidity and mortality.

## Data availability statement

The raw data supporting the conclusions of this article will be made available by the authors, without undue reservation.

## Ethics statement

The studies involving human participants were reviewed and approved by the Ethics Committees of the Medical Association of Brandenburg. The patients/participants provided their written informed consent to participate in this study.

## Author contributions

NP and TW: conceptualization, methodology, investigation, writing – original draft, and writing – review and editing. FS: writing – original draft, methodology, investigation, and validation. GL and OR: investigation and writing – review and editing. MS and TG: writing – review and editing. BS: investigation, writing – original draft, review, and editing. NB: methodology and writing – original draft. All authors contributed to the article and approved the submitted version.

## Conflict of interest

The authors declare that the research was conducted in the absence of any commercial or financial relationships that could be construed as a potential conflict of interest.

## Publisher’s note

All claims expressed in this article are solely those of the authors and do not necessarily represent those of their affiliated organizations, or those of the publisher, the editors and the reviewers. Any product that may be evaluated in this article, or claim that may be made by its manufacturer, is not guaranteed or endorsed by the publisher.

## References

[1] Ohira H Tsutsui W Fujioka Y. Are short chain fatty acids in gut microbiota defensive players for inflammation and atherosclerosis? J Atheroscler Thromb. (2017) 24:660–72. doi: 10.5551/jat.RV17006 28552897 PMC5517538

[2] Natarajan N Hori D Flavahan S Steppan J Flavahan NA Berkowitz DE. Microbial short chain fatty acid metabolites lower blood pressure via endothelial G protein-coupled receptor 41. Physiol Genomics. (2016) 48:826–34. doi: 10.1152/physiolgenomics.00089.2016 27664183 PMC6223570

[3] Pluznick JL Protzko RJ Gevorgyan H Peterlin Z Sipos A Han J. Olfactory receptor responding to gut microbiota-derived signals plays a role in renin secretion and blood pressure regulation. Proc Natl Acad Sci USA. (2013) 110:4410–5. doi: 10.1073/pnas.1215927110 23401498 PMC3600440

[4] Meyer F Seibert FS Nienen M Welzel M Beisser D Bauer F. Propionate supplementation promotes the expansion of peripheral regulatory T-cells in patients with end-stage renal disease. J Nephrol. (2020) 33:817–27. doi: 10.1007/s40620-019-00694-z 32144645 PMC7381474

[5] Demigne C Morand C Levrat MA Besson C Moundras C Remesy C. Effect of propionate on fatty acid and cholesterol synthesis and on acetate metabolism in isolated rat hepatocytes. Br J Nutr. (1995) 74:209–19. doi: 10.1079/bjn19950124 7547838

[6] Haghikia A Zimmermann F Schumann P Jasina A Roessler J Schmidt D. Propionate attenuates atherosclerosis by immune-dependent regulation of intestinal cholesterol metabolism. Eur Heart J. (2022) 43:518–33. 34597388 10.1093/eurheartj/ehab644 PMC9097250

[7] Piccioni A de Cunzo T Valletta F Covino M Rinninella E Raoul P. Gut microbiota and environment in coronary artery disease. Int J Environ Res Public Health. (2021) 18:4242. 10.3390/ijerph18084242 PMC8073779 33923612

[8] Liebisch G Ecker J Roth S Schweizer S Ottl V Schott HF. Quantification of fecal short chain fatty acids by liquid chromatography tandem mass spectrometry-investigation of pre-analytic stability. Biomol Ther. (2019) 9:121. doi: 10.3390/biom9040121 PMC6523859 30925749

[9] Kindt A Liebisch G Clavel T Haller D Hormannsperger G Yoon H. The gut microbiota promotes hepatic fatty acid desaturation and elongation in mice. Nat Commun. (2018) 9:3760. doi: 10.1038/s41467-018-05767-4 30218046 PMC6138742

[10] Vieira-Silva S Falony G Belda E Nielsen T Aron-Wisnewsky J Chakaroun R. Statin therapy is associated with lower prevalence of gut microbiota dysbiosis. Nature. (2020) 581:310–5. 32433607 10.1038/s41586-020-2269-x

