# Supplementary material for: Association of plasma propionate concentration with coronary artery disease in a large cross-sectional study
Source: Front Cardiovasc Med. 2023 Feb 1;10:1063296. doi: 10.3389/fcvm.2023.1063296 (PMC9928685; doi:10.3389/fcvm.2023.1063296)
Supplement: Supplementary file 1 [file Data_Sheet_1.PDF]

Supplemental Material

Supplemental Table 1 Propionate with regards to main risk factors for coronary artery disease

	**Propionate**	**P-value**
Males	6.25 (4.43-8.04)	**0.003**
Females	6.77 (4.92-9.23)	
Hypertension	6.26 (4.45-8.11)	**<0.001**
No Hypertension	7.82 (5.60-11.1)	
Diabetes	6.29 (4.45-8.10)	0.24
No Diabetes	6.54 (4.67-8.58)	
Current smoking	6.31 (4.16-8.67)	0.53
No current smoking	6.42 (4.68-8.44)	

Data are presented as median with interquartile range. Mann-Whitney U Test were performed. Significant values are shown in bold numbers.

Supplemental Table 2 Cardiovascular risk factors associated to propionate in univariate and multivariate linear regression

	All participants		Coronary artery disease patients	
	β (95% CI)	p	β (95% CI)	p
Age	-0.067 (-0.038 to -0.003)	**0.021**	0.028 (-0.340 to 0.783)	0.47
Sex	-0.068 (-0.944 to -0.079)	**0.022**	0.030 (-0.340 to 0.783)	0.44
BMI	0.033 (-0.017 to 0.063)	0.25	0.042 (-0.022 to 0.031)	0.28
Hypertension	-0.044 (-1.01 to 0.163)	0.15	0.045 (-0.321 to 1.238)	0.25
Diabetes	0.07 (0.092 to 1.046)	**0.024**	0.110 (0.230 to 1.226)	**0.004**
Smoking	-0.083 (-1.178 to -0.181)	**0.008**	0.001 (-0.567 to 0.585)	0.98
HDL	-0.013 (-0.017 to 0.011)	0.68	-0.021 (-0.022 to 0.013)	0.62
LDL	0.020 (-0.004 to 0.007)	0.51	0.020 (-0.005 to 0.008)	0.62
Cholesterol	0.037 (-0.002 to 0.008)	0.22	0.079 (0.00 to 0.010)	**0.048**
Triglycerides	0.063 (0.00 to 0.004)	**0.041**	0.107 (0.001 to 0.005)	**0.007**
Lipoprotein-a	-0.117 (-0.009 to -0.001)	**0.006**	-0.102 (-0.008 to 0.00)	0.06
hsCRP	-0.018 (-0.214 to 0.118)	0.57	-0.012 (-0.233 to 0.170)	0.76
HbA1c	-0.039 (-0.549 to 0.195)	0.35	0.062 (-0.175 to 0.616)	0.27

Multivariate analysis adjusted for age, gender and BMI. Data are presented as β (beta coefficient) and CI (confidence interval). P value from Pearson analysis. Significant values are shown in bold numbers. Abbreviations: BMI: body mass index, HbA1c: glycated hemoglobin A1c, HDL: high-density lipoprotein, LDL: low-density lipoprotein.

Supplemental Table 3 Description of the main cardiovascular risk factors and biomarkers based on intake of statins

	Statins	No statins	P-value
N	759	494	
Age, years	69 (61-77)	63 (54-73)	**<0.001**
Male, no (%)	539 (71)	257 (52)	**<0.001**
Hypertension, no (%)	678 (89)	351 (71)	**<0.001**
Diabetes, no (%)	256 (34)	87 (18)	**<0.001**
Current smoking, no (%)	241 (32)	119 (24)	**0.002**
BMI, kg/m^2	28 (26-32)	28 (25-32)	0.42
HDL, mg/dl	47 (39-60)	54 (44-65)	**<0.001**
LDL, mg/dl	98 (77-126)	134 (108-160)	**<0.001**
Cholesterol, mg/dl	170 (143-196)	205 (178-233)	**<0.001**
Triglyceride, mg/dl	126 (93-181)	119 (90-169)	0.21
Lipoprotein-a nmol/l	15 (6-77)	12 (6-41)	0.28
hsCRP, mg/dl	0.09 (0.0-0.62)	0.20 (0.0-0.83)	**0.02**
HbA1c, %	5.9 (5.5-6.6)	5.6 (5.3-5.9)	**<0.001**
Propionate, µM	5.76 (4.2-7.5)	6.64 (4.6-8.7)	**<0.001**

Data are presented as median with interquartile range or number (%). Statistical comparison was performed by Chi square test for categorical variables and Mann Whitney test for metric variables. Significant values are shown in bold numbers. NA - not applicable; BMI - body mass index; HbA1c - glycated hemoglobin A1c; HDL - high-density lipoprotein; hsCRP – high sensitive C reactive protein; LDL - low-density lipoprotein

Supplemental Table 4. Logistic regression analysis for detection of coronary artery disease in dependence of quartiles of propionate concentrations

		Univariate			Multivariate		
		OR	95% CI	p	OR	95% CI	p
QR 1	reference	1.00			1.00		
QR 2	vs. QR 1	0.814	0.58- 1.14	0.224	0.699	0.48- 1.01	0.059
QR 3	vs. QR 1	0.732	0.53-1.02	0.065	0.626	0.43-0.91	**0.014**
QR 4	vs. QR 1	0.472	0.34- 0.66	**<0.001**	0.448	0.31- 0.65	**<0.001**

The associations of quartiles of propionate concentrations with CAD were assessed by logistic regression using univariate and multivariate models adjusted for selected traditional cardiovascular risk factors (hypertension, diabetes, smoking), age, gender, body-mass index and intake of statins. Odds ratios and 95% confidence intervals are presented. Odds ratios for continuous variables are per 1-SD increase.
